# Grand multiparity and the possible risk of adverse maternal and neonatal outcomes: a dilemma to be deciphered

**DOI:** 10.1186/s12884-017-1508-0

**Published:** 2017-09-19

**Authors:** Ghadeer K. Al-Shaikh, Gehan H. Ibrahim, Amel A. Fayed, Hazem Al-Mandeel

**Affiliations:** 1 Obstetrics and Gynecology Department, College of Medicine, King Khalid University Hospital, King Saud University, Riyadh, Saudi Arabia; 2 Department of Medical Biochemistry, Faculty of Medicine, Suez Canal University, Round Road, Ismailia, 41511 Egypt; 3 College of Medicine, Princess Nourah Bint Abdulrahman University, Riyadh, Saudi Arabia; 4 Department of Biostatistics, High Institute of Public Health, Alexandria University, Alexandria, Egypt

**Keywords:** Grand multiparity, Maternal outcome, Neonatal outcome, Pregnancy outcome, Prenatal care, Risk

## Abstract

**Background:**

The relation between grand multiparity (GMP) and the possible adverse pregnancy outcomes is not well identified. GMP (parity ≥5 births) frequently occurs in the Arab nations; therefore, this study aimed to identify the correlation between GMP and the different adverse maternal and neonatal outcomes in the Saudi population.

**Method:**

This cohort study was conducted on a total of 3327 women from the labour ward in King Khaled University Hospital, Riyadh, Saudi Arabia. Primiparous, multiparous and grand multiparous females were included. Socio-demographic data and pregnancy complications like gestational diabetes or hypertension, preeclampsia and intrauterine growth restriction were retrieved from the participants’ files. In addition, the labour ward records were used to extract information about delivery events (e.g. spontaneous preterm delivery, caesarean section [CS]) and neonatal outcomes including anthropometric measurements, APGAR score and neonatal admission to the intensive care.

**Results:**

Primiparas responses were more frequent in comparison to multiparas and GMP (56.8% and 33%, and 10.2% respectively). In general, history of miscarriage was elevated (27.2%), and was significantly higher in GMP (58.3%, *p* < 0.01). Caesarean delivery was also elevated (19.5%) and was significantly high in the GMP subgroup (*p* < 0.01). However, after adjustment for age, GMP were less likely to deliver by CS (odds ratio: 0.6, 95% CI: 0.4–0.8; *p* < 0.01). The two most frequent pregnancy-associated complications were gestational diabetes and spontaneous preterm delivery (12.6% and 9.1%, respectively). The former was significantly more frequent in the GMP (*p* < 0.01). The main neonatal complication was low birth weight (10.7%); nevertheless, neonatal admission to ICU was significantly higher in GMP (*p* = 0.04), and low birth weight was more common in primiparas (*p* < 0.01). Furthermore, logistic regression analysis revealed an insignificant increase in the maternal or neonatal risks in GMP compared to multiparas after adjustment for age.

**Conclusion:**

Grand multiparous Saudi females have similar risks of maternal and neonatal complications compared to the other parity groups. Advanced age might play a major role on pregnancy outcomes in GMP. Nevertheless, grand multiparty might not be discouraged as long as women are provided with good perinatal care.

## Background

Grand multiparity (GMP) was defined in the older literature as giving birth seven times or higher [1]. More recent reports describe it as parity of five or more [2]. With the widespread application of family planning in developed countries, GMP has decreased in Western society and its prevalence became very low (~4% of all births) [3]. In many parts of the world, GMP is associated with higher risks of obstetric complications such as gestational diabetes, gestational hypertensive disorders [4, 5], maternal anemia, postpartum hemorrhage, congenital malformations and perinatal mortality [6]. However, other studies found a lower incidence of these complications in grand multiparous women [7]. Furthermore, obstetric risks might also be attributed to the advanced maternal age in addition to high parity. Therefore, maternal age must be examined as a confounder while interpreting the risk of maternal and neonatal complications in GMP women [6].

GMP is seen frequently in Arab nations like the Saudi population. Kumari and Badrinath [8] reported a significant increase in gestational diabetes and macrosomia in a sample of Arabic grand multiparous [8]. Therefore, GMP is expected to represent a risk factor of pregnancy related complications in Saudis as grand multiparity is still prevalent. The main point of interest for obstetricians in a case of GMP is how this might alter labour and delivery expectations, in addition to the risk of maternal morbidity and mortality [4]. Two decades ago, Fayed et al. [9] excluded obstetric risks in Saudi GMP women if they are provided with a high socioeconomic environment and receive high standard perinatal care. Later on, a scanty number of studies investigated the effect of parity on the pregnancy complications in Saudi population [10, 11], while neonatal outcomes have not been explored yet.

Grand multiparty will continue to exist in Saudi Arabia as the concept of having large families is highly accepted. Further research is needed to clarify the impact of GMP on pregnancy and neonatal outcomes as previous data are not conclusive. The current study was conducted to determine the incidence of adverse maternal and neonatal outcomes in different parity status and to evaluate the effect of GMP on these complications in Saudi females with comparison to primiparity and multiparity.

## Methods

This cohort study was designed to examine the relationship between parity and overall rates of maternal and neonatal complications. The study was conducted in accordance with the guidelines in the Declaration of Helsinki and was approved by the Institutional Review Board (IRB) of King Saud University.

### Inclusion and exclusion criteria

In the period between November 2013 and November 2014, a total of 3327 women who had singleton births were recruited from the labour ward in King Khaled University Hospital (KKUH), Riyadh, Saudi Arabia. Participants were classified into three groups according to parity: primipara [one birth], multipara [2–4 births], and grand multipara [5 or more births]. Exclusion criteria included: pregnant women with multiple gestations; illnesses that might increase the pregnancy adverse outcomes such as renal and cardiac diseases, and previous uterine scar. Females presented with any form of fetal malpresentation were also ruled out from the study. An informed verbal consent was obtained from all participants prior to their participation in the study.

### Data collection

Socio-demographic details, maternal health, and information about pregnancy, delivery and perinatal outcomes were collected from all subjects. Paper medical records were abstracted to ascertain the women’s medical status throughout gestation. Adverse pregnancy outcomes (e.g. Anemia, gestational diabetes, gestational hypertension [De novo hypertension alone after 20 weeks gestation in a previously normotensive woman], preeclampsia [new onset of hypertension after 20 weeks gestation with proteinuria (≥300 mg/24 h)], placental pathologies, intrauterine growth restriction and antepartum hemorrhage) were retrieved from their files. Delivery events (e.g. spontaneous preterm delivery [birth before 37 weeks of gestation], need for induction of labour, mode of delivery, cesarean section (CS), postpartum hemorrhage, perinatal deaths. And maternal admission to the intensive care unit [ICU]) and birth outcomes (e.g. anthropometric birth outcomes, APGAR score in the 5th minute after delivery, congenital malformations and newborn admission to the ICU) were noted after delivery. A newborn birth weight of <2500 g was considered low, in addition low APGAR score corresponded to a score < 7 in the 5th minute after delivery [12].

### Statistical analysis

Data were analyzed using the SPSS software v.20.0 for Windows® (SPSS Inc., Chicago, IL, USA). Univariate analysis and differences between groups were assessed using the one way Analysis of Variance (ANOVA), or Chi-square (χ^2^) test when appropriate. Multiple logistic regression analysis was used to adjust for the age difference among the studied groups and adjusted odds ratios were calculated for maternal and neonatal outcomes. All statistical tests were two-tailed, and a *p* value <0.05 was considered statistically significant.

## Results

During the study period, there were 3327 deliveries, out of which 341 (10.2%) were grand multiparas and the rest included primiparas and multiparas (56.8% and 33%, respectively). Table 1 shows the socio-demographic data, pregnancy and neonatal outcomes of the total study’s participants. The majority of women aged from 25 to 30 years (58.4%) and they were mostly Saudis (91.4) and housewives (85.8%). Unfortunately, positive history of miscarriage among the participants was high (27.2%). The main pregnancy-associated complications were gestational diabetes and spontaneous preterm delivery (12.6% and 9.1%, respectively). Gestational hypertensive disorders, intrauterine growth restriction and maternal admission to the ICU showed a frequency lower than 2% each. Furthermore, 19.5% of our study population delivered by CS. Neonatal complications identified in the study were low birth weight (10.7%), followed by neonatal admission to the ICU (4%), low APGAR score (1.5%) and congenital anomalies (1.3%).

**Table 1:** Socio-demographic data, medical health and reproductive information of the study population (n = 3327)

Age groups (n, %)	
< 25 years	819 (24.6)
25–35 years	1944 (58.4
> 35 years	564 (17)
Nationality (n, %)	
Saudi	3057(91.4)
Non Saudi	290(8.6)
Education (n, %)	
School	2665(79.6)
University or higher	682(20.4)
Working Status (n, %)	
Housewife	2365(85.2)
Employee	398(14.3)
Student	14(0.5)
Smoking (n, %)	84(3.0)
Gestational age at delivery (mean ± SD)	38.6 ± 2.2
BMI at delivery (Kg/m^2^; mean ± SD)	31.5 ± 6
History of miscarriage (n, %)	899 (27.2)
History of multiple pregnancy (n, %)	115 (3.4)
History of chronic diseases (n, %)	
Hypertension	34(1.0)
Diabetes mellitus	46(1.4)

Stratification of the study population according to parity showed that grand multiparous females were more likely to be of advanced age (*p* < 0.01) and to be housewives (*p* < 0.01) (Table 2). History of miscarriage was significantly higher in GMP group compared to primiparas and multiparas (*p* < 0.01). Most of the pregnancy complications were more frequent in GMP group compared to the other parity sub-groups. GMP women were more likely to have gestational diabetes (*p* < 0.01), gestational hypertension (*p* = 0.01), and ICU admission (*p* = 0.03) (Table 2). On the other hand, preeclampsia and intrauterine growth restriction were more common in primipara compared to the other parity groups, yet the difference in preeclampsia was not statistically significant (*p* = 0.07 and 0.02, respectively). Preterm delivery, the second most common pregnancy complication in the total participants, was higher in GMP group compared to primipara and multipara, yet the difference did not reach statistical significance (*p* = 0.05). In addition, the frequency of CS was higher in GMP when compared to the other parity groups (*p* < 0.01) (Table 2). Comparison of the neonatal complications in the three parity group showed that neonatal admission to ICU was significantly higher in the GMP group (*p* = 0.04), while low birth weight was more common in the primipara group (*p* < 0.01) (Table 2).

**Table 2:** Comparison of the study participants demographic data, pregnancy and neonatal outcomes according to parity

	Primipara *N* = 1889	Multipara *N* = 1097	Grand Multipara *N* = 341	*P* value
Age (years; mean ± SD)	26.3 ± 4.4	31.6 ± 4.8	38.2 ± 3.7	<0.01
Age groups:				
< 25 years	728(38.5)	85(7.7)	6(1.8)	<0.01
25–35 years	1091(57.8)	780(71.1)	73(21.4)	
> 35 years	70(3.7)	232(21.1)	262(76.8)	
Nationality				
Saudi	1734(91.8)	979(89.2)	327(95.9)	<0.01
Non Saudi	155(8.2)	118(10.8)	14(4.1)	
Education				
School	1467(77.7)	886(80.8)	294(86.2)	<0.01
University or higher	422(22.3)	211(19.2)	47(13.8)	
Working Status				
Housewife	1329(84.0)	773(85.5)	252(90.3)	0.01
Employee	240(15.2)	130(14.4)	27(9.7)	
Student	13(0.8)	1(0.1)	0(0.0)	
Smoking	45(2.8)	29(3.1)	10(3.5)	0.73
BMI at delivery (Kg/m^2^; mean ± SD)	30.4 ± 5.6	32.4 ± 6.1	34.6 ± 6.3	<0.01
Gestational age at delivery (years; mean ± SD)	38.7 ± 2.3	38.6 ± 2.0	38.4 ± 2.3	0.12
History of multiple pregnancy	59(3.1)	46(4.2)	10(2.9)	0.3
History of miscarriage	296(15.8)	406(37.3)	197(58.3)	<0.01
Pregnancy outcomes				
Gestational diabetes	174(9.3)	156(14.4)	85(25.2)	<0.01
Pre-existing hypertension	12(0.6)	14(1.3)	8(2.4)	0.02
Gestational hypertension	31(1.6)	16(1.5)	14(4.1)	0.01
Preeclampsia	25(1.3)	5(0.5)	3(0.9)	0.07
Intrauterine growth restriction	50(2.6)	16(1.5)	3(0.9)	0.02
Spontaneous preterm delivery	173(9.4)	81(7.6)	39(11.8)	0.05
Induction of labour	372(19.8)	130(11.9)	54(15.8)	<0.01
Mode of delivery				
Spontaneous delivery	1356(72.7)	838(76.8)	251(74.9)	<0.01
Instrumental delivery	173(9.3)	17(1.6)	4(1.2)	
Cesarean section	335(18.0)	236(21.6)	80(23.9)	
Maternal admission to ICU	8(0.4)	4(0.4)	5(1.5)	0.03
Neonatal outcomes				
Baby gender (male)	921(49.0)	562(51.4)	166(49.3)	0.46
Birth weight (mean ± SD)	3.0 ± 0.5	3.2 ± 0.5	3.1 ± 0.5	<0.01
Baby’s length (mean ± SD)	49.4 ± 2.6	49.6 ± 3.1	49.3 ± 2.9	0.10
Low birth weight	236(12.7)	92(8.5)	30(9.0)	<0.01
APGAR at 5 min <7	28(1.6)	15(1.4)	8(2.4)	0.42
Neonatal admission to ICU	84(4.5)	32(2.9)	19(5.6)	0.04
Congenital Anomalies	23(1.2)	16(1.5)	7(2.1)	0.45

Data are expressed as number (percentage) unless specified

Logistic regression analysis was conducted to test the risk of pregnancy and neonatal outcomes in GMP in comparison to multiparas after adjustment for age. There was an insignificant increase in the maternal or neonatal risks in GMP compared to multiparas. Fortunately, GMP were less likely to deliver by CS (OR: 0.6, 95% CI: 0.4–0.8; *p* < 0.01) (Table 3).

**Table 3:** Logistic regression analysis showing the risk of maternal and neonatal complications in GMP in the study population in reference to multiparas

	Adjusted odds ratios (95% CI)	*P* value
Pregnancy outcomes		
Gestational diabetes	1.2 (0.78–1.8)	0.4
Gestational hypertension	1.1 (0.39–2.88)	0.9
Preeclampsia	0.97 (0.17–6.62)	0.9
Intrauterine growth restriction	0.66 (0.12–3.5)	0.6
Spontaneous preterm delivery	1.5 (0.86–2.69)	0.2
Induction of labour	1.2 (0.79–1.87)	0.4
Cesarean section	0.6 (0.4–0.8)	<0.01
Maternal admission to ICU	2.3 (0.3–19.8)	0.4
Neonatal outcomes		
Neonatal admission to ICU	1.8 (0.8–4.3)	0.1
Congenital Anomalies	1.6 (0.5–5.3)	0.4
Low birth weight	1.1 (0.6–1.9)	0.9
APGAR at 5 min <7	1.2 (0.3–4.3)	0.8

Adjusted odds ratios are calculated in comparison to the reference group, multiparous women, whose odds ratios equal 1 for each variable

## Discussion

With the advancement of family planning, grand multiparity decreased tremendously in the Western countries. Though the incidence of GMP has declined in the Saudi population as well, it decreased from 29% [10] to 5.3% in a more recent study [11] and 10.2% in the current research, GMP remains frequent due to different factors. The impact of culture cannot be dismissed when considering this topic. Throughout the Middle Eastern region, India, Pakistan, and Africa, large families are highly valued and are a measure of high fertility [13]. In addition, the practice of early marriages and religious beliefs that do not support the use of contraception are considered serious challenges that cause an increase in the incidence of GMP in the Saudi population. Whether this represents an obstetric problem or not should be extensively investigated as the risk of complications is thought to be minimized in high-income countries as they provide a high quality health-care system [14]. In addition, there are few data on the relation and nature of maternal and neonatal complications with GMP, especially in Saudis.

The current study identified different pregnancy and neonatal complications in different parity groups with comparison of their prevalence and their potential risk in association with GMP. History of miscarriage was elevated in the GMPs in addition to the high prevalence of gestational diabetes, while anemias associated with pregnancy and placental pathologies were not identified. Cesarean deliveries and spontaneous preterm delivery were the most common obstetric complications in GMPs in addition to maternal admission to ICU that was highly frequent in this parity group. Moreover, neonatal admission to the ICU was more frequent in GMPs and unexpectedly low birth weight was more common in primiparas. In general, grand multiparous females had similar risk of pregnancy and neonatal complications compared to multiparas. However, it seems that GMP decreases the likelihood for CS delivery.

Different maternal and neonatal complications have been described in the literature. The more common adverse effects consistently linked to GMP were gestational diabetes, anemia, placenta previa, malpresentation, low birth weight, and increased perinatal mortality [12, 15–17]. However, it should be noted that gestational diabetes, a common pregnancy complication in this study, was more frequent in GMPs. However, in regression models controlling for age, GMP was not associated with higher risk of gestational diabetes. Similarly, Fowler-Brown et al. [18] found that the risk of diabetes in GMP was reduced after adjustment for the maternal age as well as the body mass index (BMI). The authors highlighted the effect of old age and increased BMI on the risk of diabetes mellitus (DM) development. On the other hand, GMP had a 27% increased risk of type 2 diabetes mellitus in a large cohort of Caucasian and African-American women [19]. The elevated percentage of gestational diabetes in GMP group of the current study, as well as the total participants, can be attributed to the high prevalence of DM in the general population. According to the latest WHO estimates, Saudi Arabia ranked the 2nd in the Middle East and the 7th worldwide regarding the rate of diabetes mellitus [20].

The current study showed that the rate of CS was high (~20%). This is higher than the one suggested by the WHO indicating that it should not exceed 15% [21]. Similar percentage was documented by a study conducted on another cohort of Saudi GMP females [22]. This increase in CS incidence has been attributed to several reasons. Grand multiparity was suggested as one of the main socio-demographic factors in CS decision making [23]. An interesting finding of our study is that grand multiparity favors normal delivery. Similar results were demonstrated in several studies [24–26], while few showed no difference [2] or a slight increase in CS rate [6]. Given the adverse effects of CS, obstetricians should take these data into consideration to avoid unnecessary CS in grand multiparous women.

The increase in the frequency of spontaneous preterm delivery among the study GMP women was also reported previously by Mgaya et al. [12] and Tai & Urquhart [27]. On the other hand, low birth weight was less frequent in GMP compared to other parity groups, yet these two adverse pregnancy outcomes are more likely to be related. In agreement to our results, a systemic review involving a meta-analysis of 41 studies found no association between GMP and low birth weight. The latter was significantly increased in primiparas [28]. Moreover, it should be noted that fetal growth is influenced by other variables like chronic maternal diseases, e.g. anemia, DM and hypertension [29]. Another important factor that should be considered is the maternal health, a problem that is correlated with several adverse pregnancy outcomes. Recurrent pregnancies as well as breastfeeding predispose to poor maternal nutrition [30]. These findings, in addition to the high frequency of miscarriage reported herein, might be explained by the possible fear of the physician, and also the mother, from fetal loss. It might represent an attempt for any early delivery to end the pregnancy successfully.

Factors that influence adverse maternal and neonatal outcomes should be identified through evidence-based medicine. Considering the high prevalence of GMP and the unmet need for family planning in Saudi Arabia, an intensive and adequate health services should be provided to these women to reduce the potential risk of complications. Furthermore, health education regarding weight control and healthy nutrition among GMP women with older age might help reduce the risk of possible maternal and neonatal complications. Health care providers should implement policies and design appropriate health education plans to reduce preventable maternal and neonatal complications and to improve the quality of prenatal care.

## Conclusion

To date, the findings on the association between GMP and maternal/neonatal outcomes are not conclusive. Our study showed that grand multiparous Saudi females have similar rates of maternal and neonatal complications compared to multiparous. Therefore, data on the increased risks of maternal and neonatal complications in GMP should be interpreted carefully due to the association of other confounders like the advanced maternal age, socioeconomic status and perinatal care. Accurate data on the magnitude of this obstetric problem in the Saudi population should be further explored. In addition, further study is required to investigate the possible causes of the high incidence of miscarriage detected in GMP women of this study.

## Abbreviations

BMI: Body mass index
CI: Confidence interval
CS: Cesarean section
DM: Diabetes mellitus
GMP: Grand multiparity
ICU: Intensive care unit
IRB: Institutional review board
KKUH: King Khaled University Hospital
SD: Standard deviation
